# BASIC AMINO ACID CARRIER 2 gene expression modulates arginine and urea content and stress recovery in Arabidopsis leaves

**DOI:** 10.3389/fpls.2014.00330

**Published:** 2014-07-16

**Authors:** Séverine Planchais, Cécile Cabassa, Iman Toka, Anne-Marie Justin, Jean-Pierre Renou, Arnould Savouré, Pierre Carol

**Affiliations:** ^1^Laboratory APCE, URF5, Université Pierre et Marie CurieParis, France; ^2^URGV, Department of Plant Genomics Research, CNRS/INRAEvry, France

**Keywords:** Arabidopsis, arginine, basic amino acid carrier, MCF, mitochondria, stress

## Abstract

In plants, basic amino acids are important for the synthesis of proteins and signaling molecules and for nitrogen recycling. The Arabidopsis nuclear gene *BASIC AMINO ACID CARRIER 2* (*BAC2*) encodes a mitochondria-located carrier that transports basic amino acids *in vitro*. We present here an analysis of the physiological and genetic function of BAC2 *in planta*. When *BAC2* is overexpressed *in vivo*, it triggers catabolism of arginine, a basic amino acid, leading to arginine depletion and urea accumulation in leaves. *BAC2* expression was known to be strongly induced by stress. We found that compared to wild type plants, *bac2* null mutants (*bac2-1*) recover poorly from hyperosmotic stress when restarting leaf expansion. The *bac2-1* transcriptome differs from the wild-type transcriptome in control conditions and under hyperosmotic stress. The expression of genes encoding stress-related transcription factors (TF), arginine metabolism enzymes, and transporters is particularly disturbed in *bac2-1*, and in control conditions, the *bac2-1* transcriptome has some hallmarks of a wild-type stress transcriptome. The BAC2 carrier is therefore involved in controlling the balance of arginine and arginine-derived metabolites and its associated amino acid metabolism is physiologically important in equipping plants to respond to and recover from stress.

## Introduction

The Arabidopsis BASIC AMINO ACID CARRIER 2 (BAC2) protein belongs to the plant mitochondrial carrier family (MCF), a diverse group of proteins with a wide range of transport selectivities and functions (Picault et al., 2004; Haferkamp, 2007; Haferkamp and Schmitz-Esser, 2012). BAC2 is in the sub-family of BAC eukaryotic mitochondrial basic amino acid carriers, which have broadly similar biochemical functions of mitochondrial transport of arginine, ornithine, and sometimes citrulline (Palmieri et al., 1997, 2006a,b, 2011; Palmieri and Pierri, 2010). The yeast *arg11* mutant is deficient in arginine synthesis because ornithine is not exported from mitochondria (Crabeel et al., 1996; Palmieri et al., 1997; Soetens et al., 1998). *BAC* from fungi and animals and *BAC1* and *BAC2* from Arabidopsis all complement the *arg11 S. cerevisiae* mutant, replacing the function of the yeast ARG11 arginine and ornithine carrier (Catoni et al., 2003; Fiermonte et al., 2003; Hoyos et al., 2003). Despite similar transport functions, the biological role of BAC in different eukaryotes is different because the physiology of organisms is different. The phenotypes of BAC mutants can be studied to assign physiological roles according to the organism's particular metabolism and anatomy. For example, in humans mutations in the mitochondrial arginine and ornithine carrier SLC25A15/ORNT1 (Fiermonte et al., 2003) lead to hyperornithinemia-hyperammonemia-homocitrullinemia (HHH) syndrome which may be fatal. Cells accumulate excess ornithine, ammonia, and citrulline because the lack of ornithine import into mitochondria disrupts the urea cycle (Camacho et al., 1999; Palmieri, 2008).

What effect does mutation of a BAC mitochondrial carrier have on plant metabolism? The effect is unlikely to be as severe as in HHH syndrome since neither ornithine nor arginine is synthesized in the mitochondria, rather in plastids from glutamate (Funck et al., 2008). However, the transport of arginine to the mitochondria in plants is important because arginine is a protein building block, a metabolic precursor of other amino acids, and a form in which nitrogen is assimilated. For example, arginine degradation in mitochondria leads to the formation of ornithine and urea via arginase activity (for review see Slocum, 2005). In turn, ornithine can be used in glutamate synthesis and urea is a source of nitrogen that can be sensed by the plant cell and used for amino acid synthesis (Mérigout et al., 2008). A specific role for mitochondrial transport of basic amino acid in plants may be to recycle nitrogen when large amounts of nitrogen is needed for growth, e.g., during germination (Catoni et al., 2003; Hoyos et al., 2003; Taylor et al., 2010). Arginine and/or urea dependent recycling is also important for metabolism in source/sink plant organs and under many kinds of stress (Witte, 2011). In other photosynthetic organisms, such as the marine unicellular diatom *Phaeodactylum tricornutum*, a diverted urea cycle, which includes arginine and its catabolites ornithine and urea, is important for nitrogen assimilation and recycling (Allen et al., 2011).

There are three BAC-related genes in Arabidopsis. *BAC1* (At2g33820) and *BAC2* (At1g79900) genes encode basic amino acid carriers as confirmed in *in vitro* assays (Catoni et al., 2003; Hoyos et al., 2003; Palmieri et al., 2006b). Arabidopsis A BOUT DE SOUFFLE (BOU, At5g46800) is phylogenetically related to other BAC and is similar to carnitine carriers from yeast and animals (Lawand et al., 2002; Picault et al., 2004; Toka et al., 2010). However, the *in vitro* transport function of BOU has not been determined. Based on their gene expression patterns, BOU, BAC1, and BAC2 are predicted to have separate physiological roles. BOU expression is regulated by light and is involved in CO_2_ assimilation and photorespiration (Lawand et al., 2002; Eisenhut et al., 2013). BAC1 is likely to be involved in storage remobilization after germination in Arabidopsis and rice (Catoni et al., 2003; Hoyos et al., 2003; Taylor et al., 2010). BAC2 is expressed in response to stress, especially hyperosmotic stress, and during senescence (Catoni et al., 2003; Hoyos et al., 2003; Toka et al., 2010).

The goal of this study was to investigate the importance of the stress-induced BAC2 mitochondrial arginine carrier in Arabidopsis. *BAC2* was over-expressed to analyze its role in arginine metabolism *in planta*. The physiological role of BAC2 was also investigated by measuring the ability of mutant and wild-type plants to recover from hyperosmotic stress. The importance of BAC2 in genetic regulation in the stress response was also observed in the *bac2-1* mutant transcriptome. Overall our results show that arginine transport via BAC2 indirectly modulates expression of genes coding for transcription factors (TF), nitrogen and arginine metabolic enzymes, and other transporters.

## Materials and methods

### Cloning of *BAC2* for overexpression *in planta*

The pBG5 vector is a binary vector containing the *BAC2* cDNA downstream of the 35S promoter and upstream of the *GFP* gene (Toka et al., 2010). pBG5 was digested with SalI and a stop codon was introduced at the end of *BAC2* cDNA by ligating the digested vector to annealed oligonucleotides BOUL-STOP-XBA-R and BOUL-STOP-XBA-F (see Toka et al., 2010 for primer sequences). The construct was checked by sequencing then used to transform wild-type or *bac2-1* mutant plants. Transgenic plants were genotyped by PCR on genomic DNA using primers BOULA (5′-GGTGAGCAAGAGGCTCTGTA-3′) and BOULATG2 (5′-CTCTAGAATGGATTTCTGGCCGGAG-3′).

### Semi-quantitative RT-PCR

RNA was extracted from 100 mg of seedlings with the RNeasy Plant Mini kit (Qiagen) including DNase treatment during purification. Reverse transcription and subsequent PCR was performed as described in Toka et al. (2010).

### Arabidopsis lines and culture conditions

*Arabidopsis thaliana* Heynh. Col-0 was used as the wild type (WT) and compared to the homozygous *bac2-1* KO mutant line, which is a null mutant in the Col-0 background (Toka et al., 2010). Seeds were surface-sterilized using a mixture of sodium hypochloride-ethanol (20:80, v/v) for 10 min, rinsed once in ethanol and dried. Seeds were imbibed in sterile 0.5 × MS medium (Murashige and Skoog, 1962) overnight at 4°C to synchronize germination. Seeds were grown at 22°C under continuous light (90 μmol m^−2^ s^−1^) on solid 0.5 × MS medium with 0.8% agar (pH 5.8). To trigger hyperosmotic stress, mutant and WT plants were grown on grids and then transferred onto 0.5 × MS solution containing 1% saccharose with 0.4 M mannitol for 24 h. Mannitol was omitted for controls. When grown on soil, plants were placed in a culture chamber (22°C, 50% humidity, 16 h light) in individual pots containing a vermiculite-perlite-soil mix (1:1:1, vol/vol/vol, Tref substrate GV01). To measure the growth response during stress, plants were grown on vertical plates containing solid medium with concentrations of mannitol increasing from 0 to 0.3 M. Roots were monitored every day and photographed, then the root length was measured from digital images using ImageJ software. To assess stress recovery, 8-day-old seedlings were subjected to 24 h of hyperosmotic stress then rinsed with water and placed on soil for 1 week. The first two leaves of at least 30 seedlings per sample were placed flat on moist filter paper then photographed. Leaf area was measured from digital images using ImageJ software.

### Amino acid analysis

Amino acid analysis was done at the INRA facilities in Versailles (France) (http://www7.versailles-grignon.inra.fr/green_chemistry_platform_eng/) from 100 mg of frozen plant tissues ground in liquid nitrogen. Total free amino acids were extracted in a solution of 2% 5-sulfosalicylic acid as described by Toka et al. (2010) and Ferrario-Méry et al. (1997).

### Extraction and quantification of urea

Urea was extracted from 100 mg of fresh or frozen plant tissue and quantified according to the method of Mérigout et al. (2008) adapted from Killingsbaeck (1975). Tissue was ground in 1 mL of 10 mM formic acid then 30 μL of the extract was added to 1 mL of H_2_O-acid reagent-color reagent (1:1:1, v/v/v) where the acid reagent was 20% (v/v) H_2_SO_4_, 0.06% (v/v) 74 mM ferric chloride hexahydrate in 9% (v/v) orthophosphoric acid, and the color reagent was 7% (v/v) 0.2 M diacetylmonoxime, 7% (v/v) 0.05 M thiosemicarbazide. The samples were incubated for 15 min at 98°C then cooled for 5 min on ice.

Urea concentration was determined by comparing the absorbance of the sample at 540 nm to the absorbance of urea dilutions ranging from 5 to 0.05 mM in 10 mM formic acid.

### Urease activity

Urease activity was determined as described in Witte and Medina-Escobar (2001) except that DTT was not used and the gel filtration step was omitted. Freshly harvested Arabidopsis tissues (100 mg) were used to prepare the reaction samples.

### Transcriptome analysis

Microarray analysis was carried out at the Unité de Recherche en Génomique Végétale (Evry, France) using CATMA chipscontaining 24,576 gene-specific tags corresponding to 22,089 Arabidopsis genes (Crowe et al., 2003; Hilson et al., 2004). Plants were collected at the 1.0 growth stage (Lurin et al., 2004). RNA samples were extracted from 8-day-old seedlings with the Qiagen RNeasy kit. For each comparison, one technical replicate with fluorochrome reversal was performed for each biological replicate (i.e., four hybridizations per comparison). The labeling of cRNAs with Cy3-dUTP or Cy5-dUTP (Perkin-Elmer-NEN Life Science Products), hybridization and scanning were performed as described in Lurin et al. (2004).

### Data deposition

Microarray data from this article were deposited in the Gene Expression Omnibus (http://www.ncbi.nlm.nih.gov/geo/) under accession number GSE15063 [*bac2-1* mutant transcriptome modification during osmotic stress (*Arabidopsis thaliana*)] and in CATdb (http://urgv.evry.inra.fr/CATdb/) as Project RS08-05_BAC2 according to “Minimum information about a microarray experiment” standards.

### Statistical analysis of microarray data

Normalization and statistical analysis was based on two dye swaps per comparison (i.e., four arrays each containing 24,576 GSTs and 384 controls) as described in Gagnot et al. (2008). The methods are available in the R package “Anapuce” (http://cran.r-project.org/web/packages/anapuce/index.html). First, one normalization per array was performed to remove systematic biases without background subtraction. Next, a global intensity-dependent normalization was performed using the lowess procedures (Yang and Thorne, 2003) to correct dye bias. Finally, for each block, the log-ratio median calculated over the values for the entire block was subtracted from each individual log-ratio value to correct for effects on each block, as well as potential print-tip, washing and/or drying effects. To determine which genes were differentially expressed from dye swap data, a paired *t*-test was performed on the log2 ratios, with a common variance for all the genes (H homoscedasticity), leading to a robust estimation of the variance and a high power of the test. Spots with extreme variance or genes for which only one observation was available were excluded. The raw *P*-values were then adjusted by the Bonferroni method, which controls the family-wise error rate (FWER) (Ge et al., 2003). We considered the genes with a Bonferroni *P*-value ≤ 0.05 as being differentially expressed, as described in Gagnot et al. (2008).

### Microarray data analysis

The list of differentially expressed genes was analyzed further. Upregulated and downregulated genes were ranked in an Excel spreadsheet according to their expression ratios. The number of genes in each cluster was visualized by using Venny software online (http://bioinfogp.cnb.csic.es/tools/venny/index.html). Gene annotations were found on the TAIR website (www.arabidopis.org) and genes with related functions were grouped together in a table. Gene ontology (GO) term finder software was used to find biological function of genes (Boyle et al., 2004) (http://amigo.geneontology.org/cgi-bin/amigo/term_enrichment).

## Results

### *BAC2* regulates arginine catabolism *in vivo*

**Figure 1:**
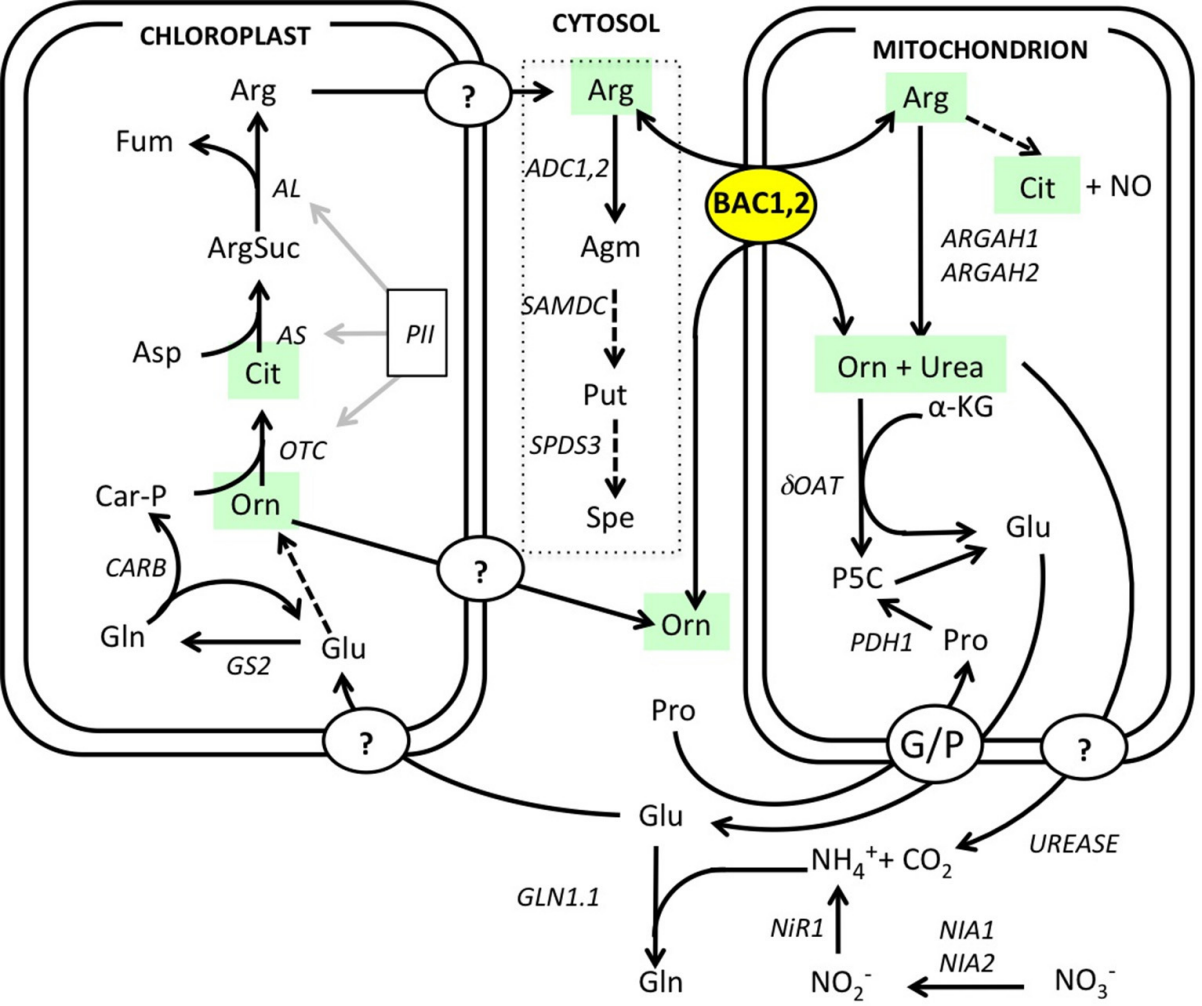
**Overview of arginine mitochondrial transport and metabolism**. Arginine metabolism map drawn according to Slocum (2005), Funck et al. (2008), and Witte (2011). Polyamine biosynthesis pathway is outlined (dotted line). Dotted arrows represent pathways with additional steps that are not detailed here. Enzyme and protein abbreviations and references: ADC, arginine decarboxylase (ADC1 At2g16500, ADC2 At4g3471); AL, argininosuccinate lyase (At5g10920); AS, argininosuccinate synthase (At4g24830); ARGAH, arginase (ARGAH1 At4g08900, ARGAH2, At4g08870); BAC, basic amino acid carriers BAC1 (At2g33820) and BAC2 (At1g79900); GS, glutamine synthetase (GS2, At5g35630); GLN, glutamine synthetase (GLN1.1 At5g37600); NIA, nitrate reductase (NIA1 At1g77760, NIA2 At1g37130); NiR1, nitrite reductase (At2g15620); dOAT, delta ornithine amino transferase (At5g46180); OTC, ornithine carbamoyl phosphate transferase (At1g75330); PII, nitrogen sensing protein (At4g01900); PRODH1, proline dehydrogenase (At3g30775); SAMDC, S-adenosine methionine decarboxylase (At3g02470); SPDS, spermidine synthase (SPDS3 At5g53120); urease (At1g67550). Metabolite abbreviations: Agm, agmatine; Arg, arginine; ArgSuc, argininosuccinate; Car-P, carbamoyl phosphate; Cit, citrulline; Fum, fumarate; Gln, glutamine; Glu, glutamate; αKG, alpha-ketoglutarate; NO, nitric oxide; Orn, ornithine; Pro, proline; Put, putrescine; P5C, delta1-pyrroline-5-carboxylate; Spe, spermidine.

**Figure 2:**
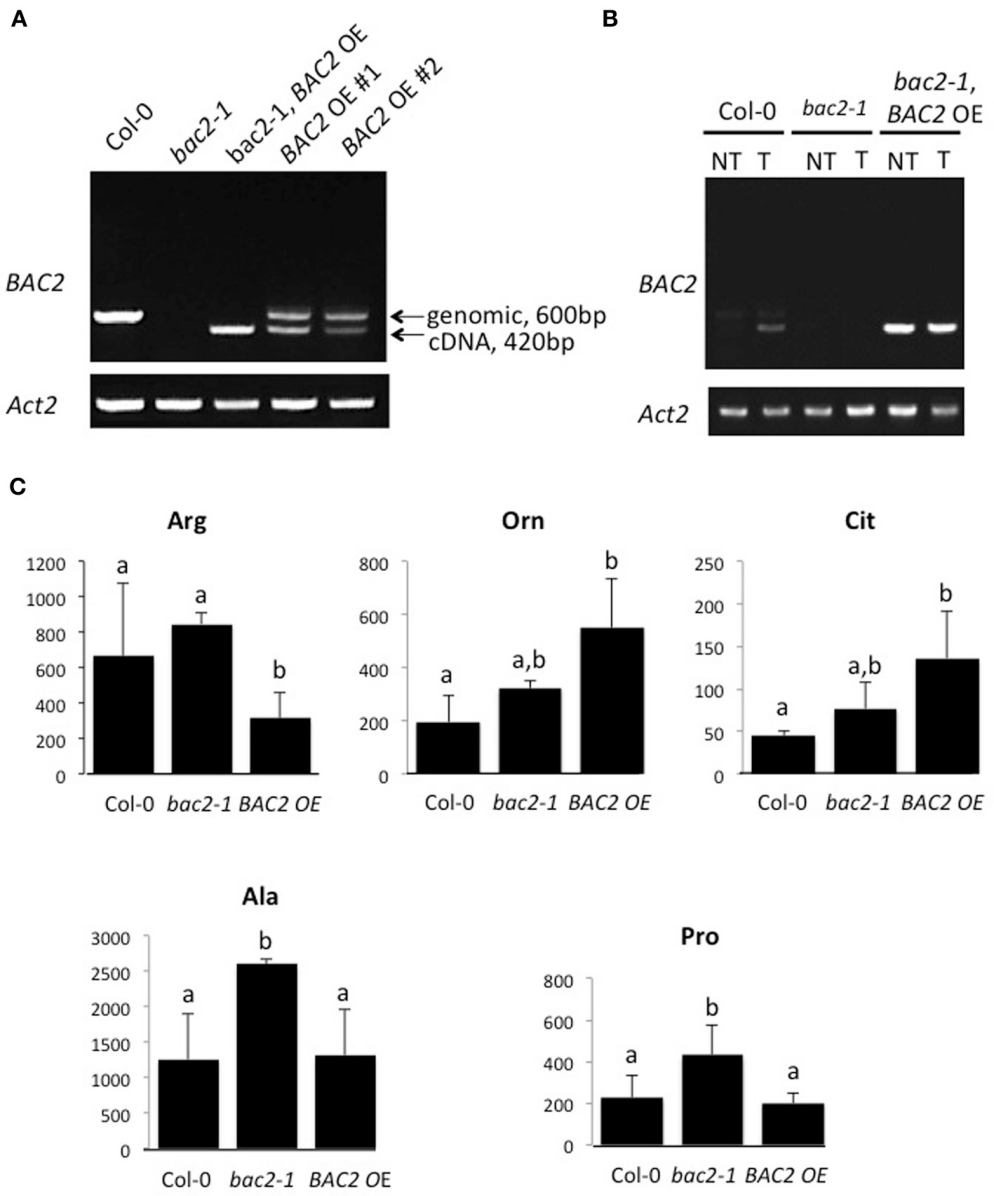
***BAC2* overexpression and amino acid content of 8-day-old Arabidopsis seedlings. (A)** PCR on genomic DNA of Arabidopsis Col-0, *bac2-1*, p35S:BAC2 in *bac2-1* genetic background (named *bac2-1, BAC2-OE*) p35S:BAC2 in Col-0 (named *BAC2-OE* #1 and *BAC2-OE* #2). Arrows indicate *BAC2* genomic DNA and cDNA sizes. ACT2 was used as a control of genomic DNA quality. **(B)** Semi-quantitative RT-PCR. *BAC2* expression levels were detected in Arabidopsis Col-0, *bac2-1*, and p35S:BAC2 in *bac2-1* (named *bac2-1, BAC2-OE*) seedlings non-treated (NT) or treated with 0.4 M mannitol (T). *ACT2* gene expression level was used as a control. **(C)** Values in nanomoles of amino acid per gram of fresh weight of total plant tissue. Col-0 is the WT control, *bac2-1* is the KO mutant, and *BAC2-OE* is an overexpressor line (*BAC2-OE* #2). Only amino acids which differ significantly between BAC2-OE and Col-0 are shown. Arg, arginine; Cit, citrulline; Orn, ornithine; Pro, proline; Ala, alanine. Letters refer to statistically different values significant at *p* < 0.05 (*n* = 3) in the Student *t*-test.

Arginine metabolism in Arabidopsis plants is under the control of synthetic and catabolic enzymes localized in different organelles (Figure 1). As a mitochondrial carrier BAC2 might contribute to regulating arginine levels in the cytosol and the mitochondria. If BAC2 has a regulatory role controlling how much arginine enters mitochondria, then changes in BAC2 abundance would be expected to alter the amounts of arginine and other amino acids in the cell. To test the hypothesis that arginine content is modulated according to the level of *BAC2* expression we engineered transgenic wild type Col-0 Arabidopsis (WT) and *bac2-1* (Toka et al., 2010) plants to constitutively express *BAC2* cDNA under the control of the strong *35S CaMV* promoter (Figure 2A). In WT, *BAC2* mRNA is expressed at a very low level in vegetative tissues (Toka et al., 2010) and *BAC2* mRNA was not detected in *bac2-1* plants. Plants harboring the *p35S:BAC2* construct expressed *BAC2* constitutively (Figure 2B) and are from now on referred to as *BAC2* overexpressor lines (*BAC2*-*OE*). The consequence of *BAC2* expression on the accumulation of arginine and other amino acids was assessed in 8-day-old plantlets. *bac2-1* plantlets contained significantly more alanine and proline than WT, but otherwise the amino acid content was quite similar (Figure 2C). The arginine and arginine-related amino acid content of *BAC2-OE* plants was very different. *BAC2-OE* plants contain less arginine than WT but more ornithine and citrulline (Figure 2C). This result was interesting as ornithine and citrulline are two products of arginine catabolism. The level of *BAC2* expression modulates arginine accumulation *in planta*.

**Figure 3:**
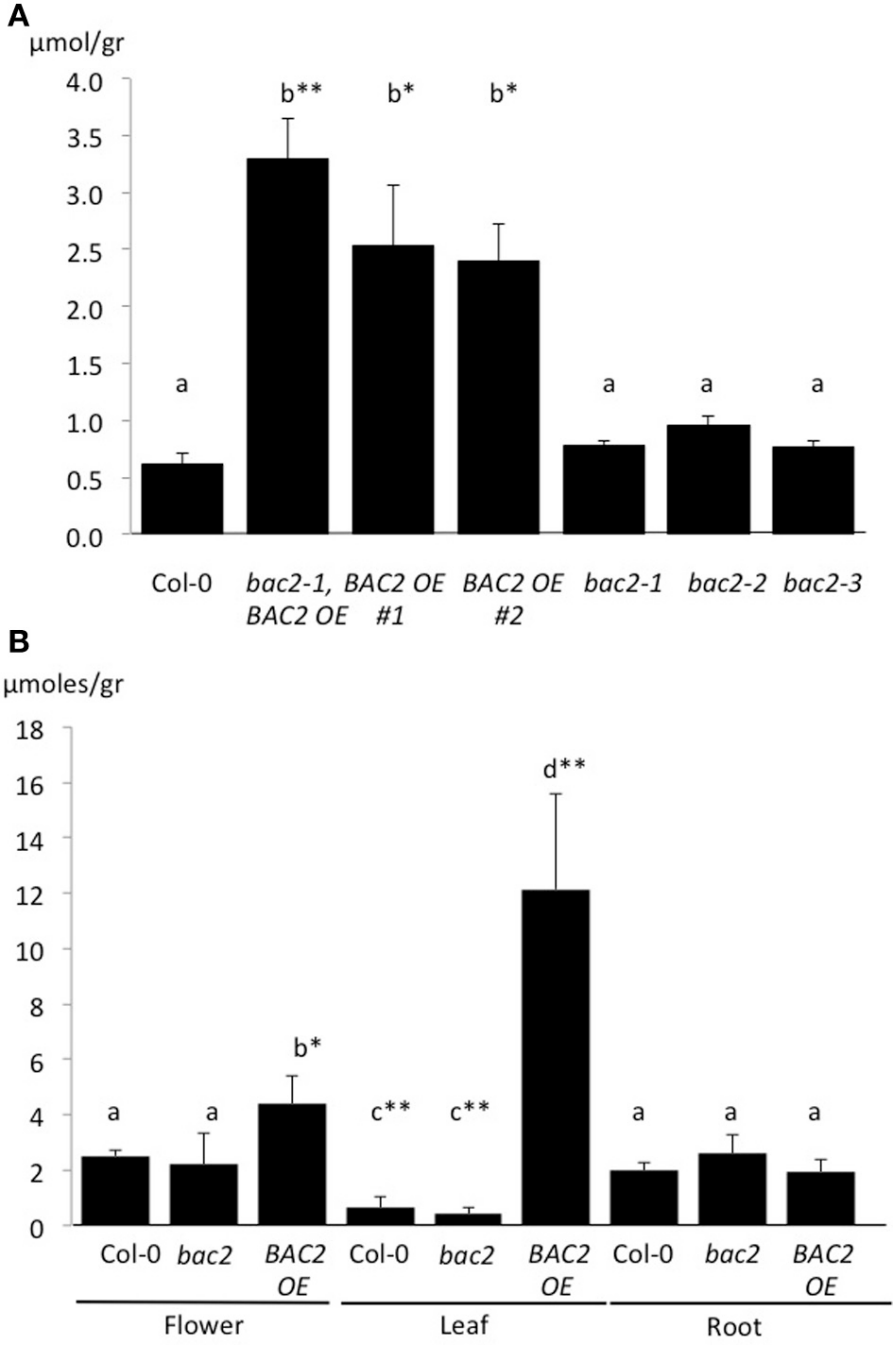
**Urea content in 8-day-old Arabidopsis seedlings. (A)** Values are given in micromoles per gram of fresh weight of total plant tissue from 8-day-old seedlings **(A)** or isolated organs in 3 week-old-plants **(B)**. Different genotypes tested are: WT (Col-0), *bac2* mutants (*bac2*-1, *bac2*-2, and *bac2*-3) and *BAC2* overexpressing lines (*BAC2-OE #2* and *bac2*-1, *BAC2-OE*). Letters refer to statistically different values that are significant ^*^p < 0.05 or highly significant ^**^p < 0.005 in the Student *t*-test (*n* = 3).

Increased arginine transport to the mitochondria via BAC2 may increase arginine availability for catabolism by mitochondrial arginases. These mitochondrial-located enzymes can metabolize arginine into urea and ornithine (Figure 1; Flores et al., 2008; for review, Witte, 2011). We reasoned that the relatively low arginine and higher ornithine contents in *BAC2*-OE lines might be a result of increased arginine catabolism. If so, then we would also expect urea content to be higher. To test this, we measured the urea content of 8-day-old seedlings of three independent *BAC2*-OE lines and three independent *bac2* mutant alleles, the *bac2-1* KO insertion and point mutations *bac2-2* and *bac2-3* (Toka et al., 2010). While WT and *bac2* mutants contain similar amounts of urea, *BAC2*-*OE* seedlings accumulate four times more urea than the other genotypes (Figure 3A). The urea content of leaves, roots and flowers was determined in adult plants (Figure 3B). WT plants accumulate urea mostly in flowers and in roots with less than 20% of the total urea present in leaf tissues (Figure 3B). *BAC2*-OE plants accumulate about 10-fold more urea in leaves compared to WT (Figure 3B). In *BAC2-OE*, urea in leaves accounts for more than 60% of the plant's total urea (Supplementary figure 1). To summarize, an increase in BAC2 expression causes an increase in the urea content of leaves.

### Nickel addition leads to urea degradation

The urea accumulation observed could result from an increase in arginine catabolism or a decrease in urea catabolism. Urea catabolism in plants depends upon the activity of urease, a nickel-requiring enzyme that catabolizes urea into ammonium and CO_2_ (Witte, 2011). Urease activity was found to be similar in all genotypes tested, suggesting that the rate of urea catabolism is similar in all the samples. However, urease activity was found to be higher when the plant growth medium was supplemented with nickel (Ni). In these conditions with Ni, urea levels in *BAC2-OE* lines were reduced to the wild-type level (Supplementary figure 2). Therefore, in our experimental conditions when Ni is a limiting factor for endogenous urease activity, arginine catabolism rates are dependent on the expression levels of *BAC2*. Urea and ornithine, both arginine metabolites, can accumulate in Arabidopsis when BAC2 is highly expressed because urease activity is low.

### BAC2 is important for recovery from hyperosmotic stress

**Figure 4:**
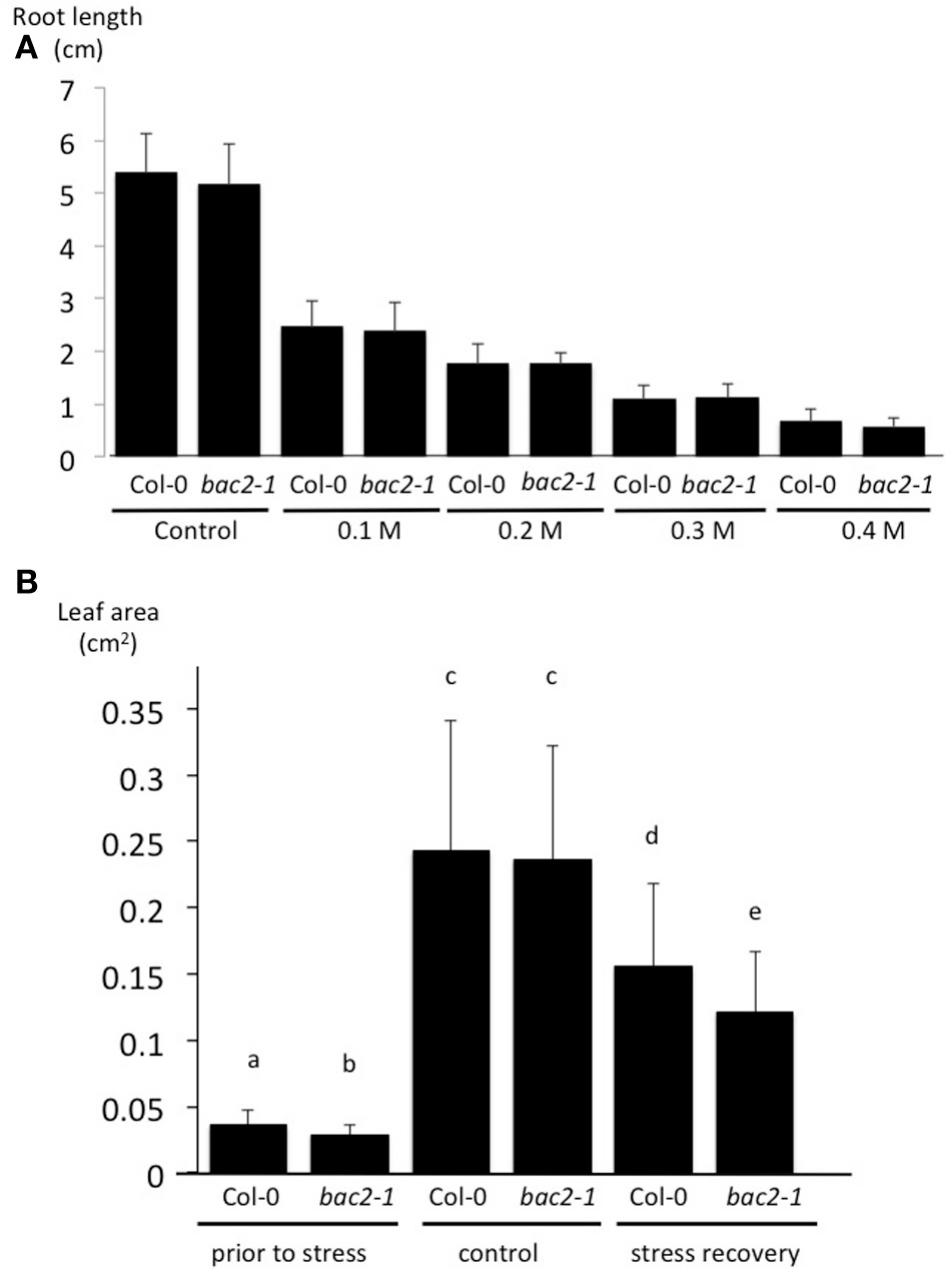
**Effect of stress on root length and leaf area growth of Arabidopsis plants**. **(A)** Measurement of Col-0 and *bac2-1* root length 9 days after sowing on increasing concentrations of mannitol to trigger an hyperosmotic stress. **(B)** The area of the first two leaves of 8-day-old Arabidopsis was measured prior to any treatment and 1 week after transfer to soil in either non-stressed control plants or stressed plants (24 h on 0.4 M mannitol). Col-0, wild type Arabidopsis; *bac2-1*, KO mutant *bac2-1*. Letters refer to statistically different values with significance of *p* < 0.05 (*n* > 40) in the Student *t*-test.

Knowing that BAC2 is expressed in Arabidopsis in response to hyperosmotic stress we wanted to investigate its physiological role in more detail. First we measured WT and *bac2-1* root growth on medium containing concentrations of mannitol up to 0.4 M, a way of inducing hyperosmotic stress. We found no significant difference in root growth between WT and mutant plants in these conditions (Figure 4A). In Arabidopsis growth can restart after hyperosmotic stress has been alleviated, in what is called “stress recovery.” After subjecting plants to 24 h of hyperosmotic stress, they were allowed to grow for 1 week on soil without stress. Leaf area was measured as a growth parameter. The area of WT leaves recovering from stress is 67% of the leaf area of the non-stressed WT control. The leaf area of *bac2-1* recovering from stress is 51% of the leaf area of the non-stressed *bac2-1* control (Figure 4B). Mutant seedlings recovering from stress have significantly reduced leaf growth (Figure 4B) suggesting that BAC2 is physiologically important in the growth recovery process.

### General and hyperosmotic stress gene expression are deregulated in *bac2-1*

**Figure 5:**
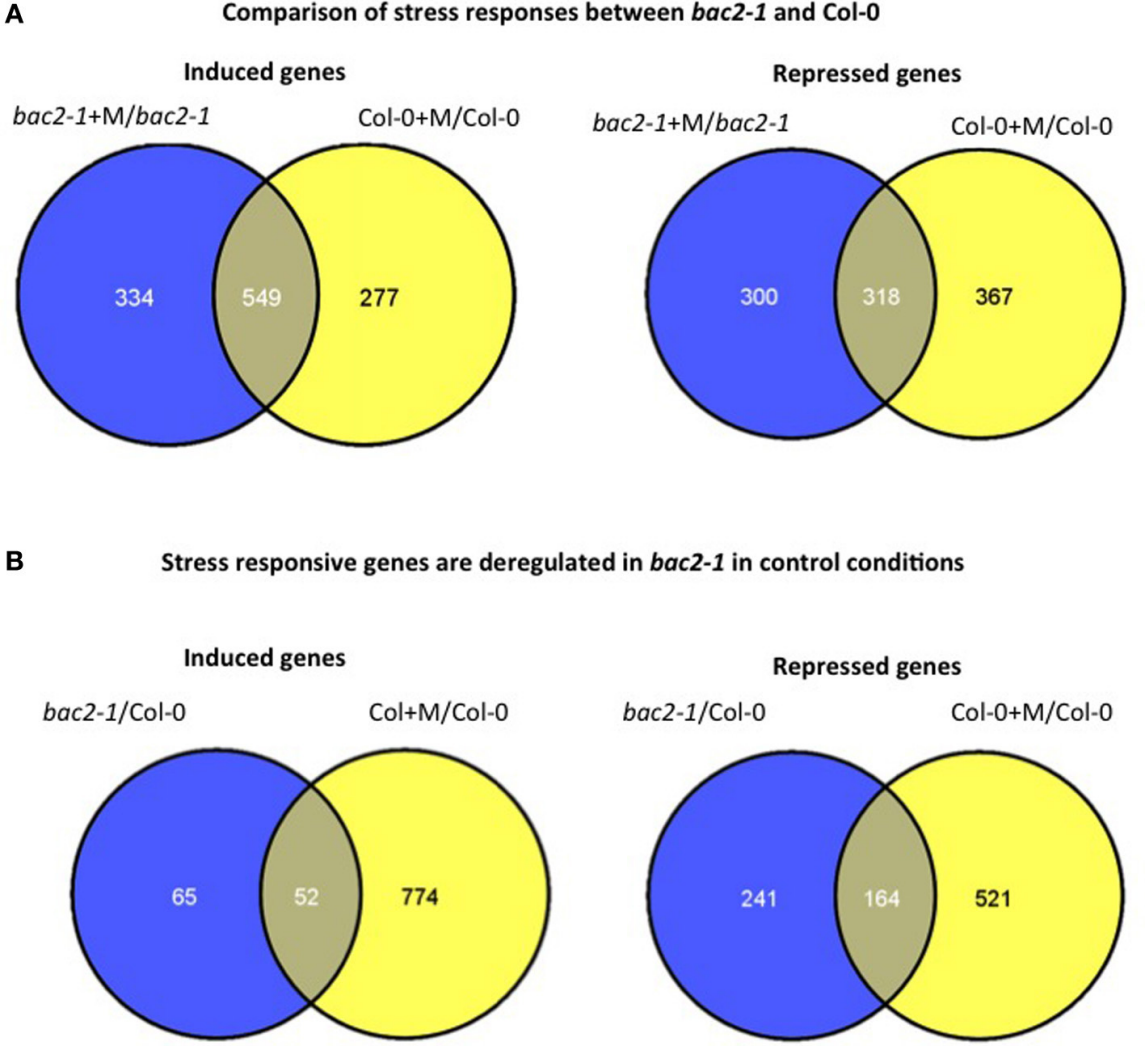
**Comparison of gene expression in *bac2-1* and Col-0 plantlets. (A)** Venn diagram of differentially expressed genes in *bac2-1* and Col-0 after water stress (+M) including induced genes or repressed genes. **(B)** Venn diagram of differentially expressed genes in *bac2-1* without stress and Col-0 after stress (+M) including induced genes or repressed genes. Venn diagrams were generated according to transcriptome data from Geosite GSE15063 and online software http://bioinfogp.cnb.csic.es/tools/venny/index.html.

We surveyed global gene expression using microarrays, comparing the transcriptomes of *bac2-1* and WT both in control conditions and after 24 h of hyperosmotic stress. After hyperosmotic stress the expression of many genes was altered as expected. Genes that are usually induced in response to hyperosmotic stress in Arabidopsis, such as *RD22*, *ERD1*, and *RD29A*, were among the genes induced both in *bac2-1* and WT (Seki et al., 2002; Skirycz et al., 2010). Overall, 867 stress-regulated genes were found to be similarly regulated in the mutant and WT. As shown in Venn diagrams in Figure 5A, *bac2-1* and WT had 549 induced genes and 318 repressed genes in common.

Overall however both in normal growth conditions and after hyperosmotic stress, the *bac2-1* transcriptome differs significantly from the WT transcriptome. Many genes are not regulated in the same way in *bac2-1* and WT. First, 334 genes were induced after stress in *bac2-1* but not in WT, and conversely 277 genes were not induced in *bac2-1* even though they were induced after stress in WT (Figure 5A). Second, the sets of genes that were repressed after stress also differ. In the *bac2-1* transcriptome, 300 genes were repressed by stress while 367 others were repressed only in WT (Figure 5A). We then considered whether the *bac2-1* transcriptome in the absence of stress has some hallmarks of a stress transcriptome. The expression of 241 genes was repressed and the expression of 65 genes was induced in unstressed *bac2-1* compared to WT in hyperosmotic stress (Figure 5B). As depicted in Figure 5B, 52 and 164 genes respectively are up or down regulated in common in *bac2-1* without stress and in WT in hyperosmotic stress. This means that BAC2-1 is needed in control conditions to regulate expression of a subset of stress-responsive genes.

### Transcription factors which are differentially expressed in *bac2-1*

Since the *bac2-1* mutation leads to a complex transcriptome modification, we reasoned that changes in gene expression could be due to a modification of TF gene expression in *bac2-1*. A global analysis of GO term enrichment revealed which TF genes are differentially expressed between *bac2-1* and the WT (Table 1). We found that 16 of the 23 TF genes affected were described as being related to abiotic stress responses. Some TF genes behave in *bac2-1* in control conditions as they would in WT after hyperosmotic stress (Table 1) giving the impression that these genes are somehow “pre-regulated” prior to stress in the mutant. The predominant biological processes in which these genes are involved are in responses to ethylene (genes encoding ERF5, SZF1, and WRKY40), salicylic acid (genes encoding WRKY33, ERF11, CZF1, and WRKY40) and biotic stress (genes encoding WRKY33, CZF1, WRKY40, ERF5, and SZF1). Deregulation of these genes in *bac2-1* in control conditions might regulate the expression of downstream target genes, thus mimicking a stress response (Figure 5B). One gene behaves quite differently. EIL1, a plant ethylene response TF that is also up-regulated by low nitrogen (Zheng et al., 2013), was downregulated in *bac2-1* in control conditions but up-regulated after hyperosmotic stress. Altogether our results show that in the absence of *BAC2* function, expression of TFs responding to environmental stresses might cause a general shift in the transcriptome, priming it for stress.

**Table 1:**
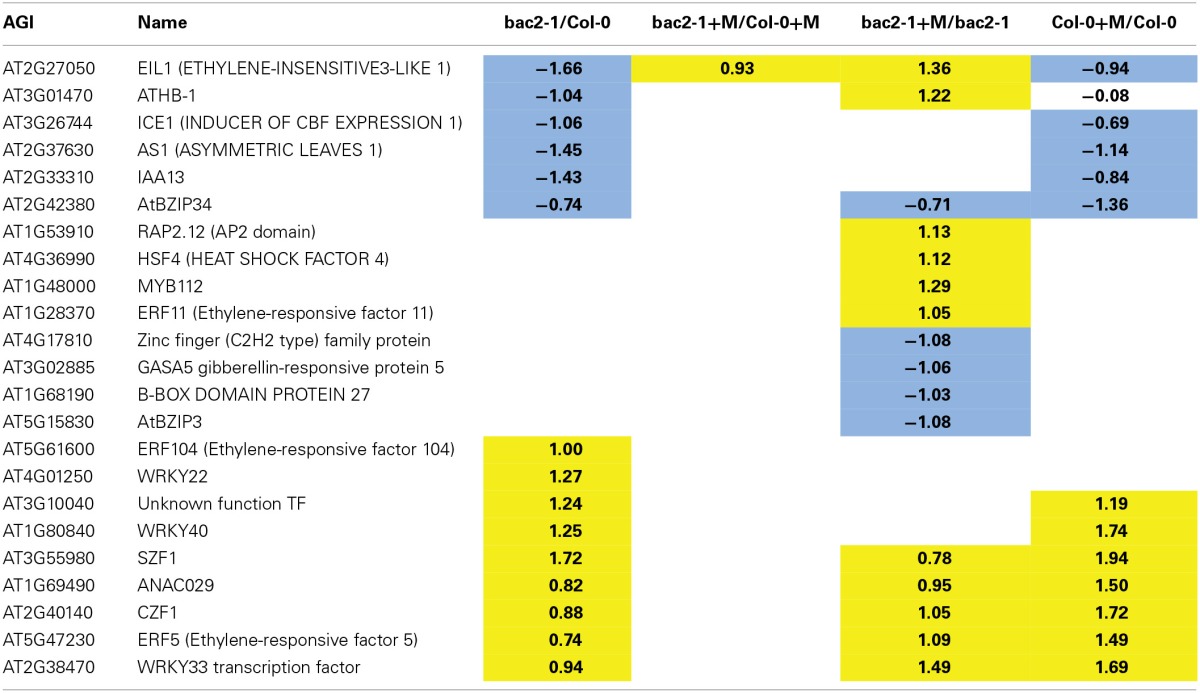
**Transcription factor genes differentially expressed in *bac2-1***.

*Detailed list of TF genes that are differentially expressed in bac2-1. Negative log2 ratios are shown on a blue background, positive ones on a yellow background. Biological functions of genes were determined by amiGO Gene Ontology Software (Boyle et al., 2004)*.

#### Nitrogen assimilation, amino acid metabolism, and transport is deregulated in *bac2-1*

To understand if the complex gene regulation seen in *bac2-1* has specific consequences in arginine relations, we extracted from the gene list any gene encoding proteins that are directly related to arginine metabolism or indirectly related like polyamine metabolism and nitrogen assimilation. (Figure 1, Table 2). Genes whose expression is significantly different in control and stress conditions include the regulatory protein *PII*, the glutamine synthesis enzyme *GS2*, and enzymes involved in polyamine biosynthesis from arginine (*ADC1* and *SAMDC1*) and nitrate reduction (*NIA2*) (Table 2). However, other genes in the same pathways behave similarly in *bac2-1* and in WT. Such genes include *ADC2* and *SPDS3*, encoding enzymes involved in polyamine biosynthesis from arginine, and *delta-OAT*, encoding a gene product that uses ornithine for proline biosynthesis. All of this information suggests that the expression of some but not all arginine-related metabolism genes is specifically altered in *bac2-1* plants.

**Table 2:**
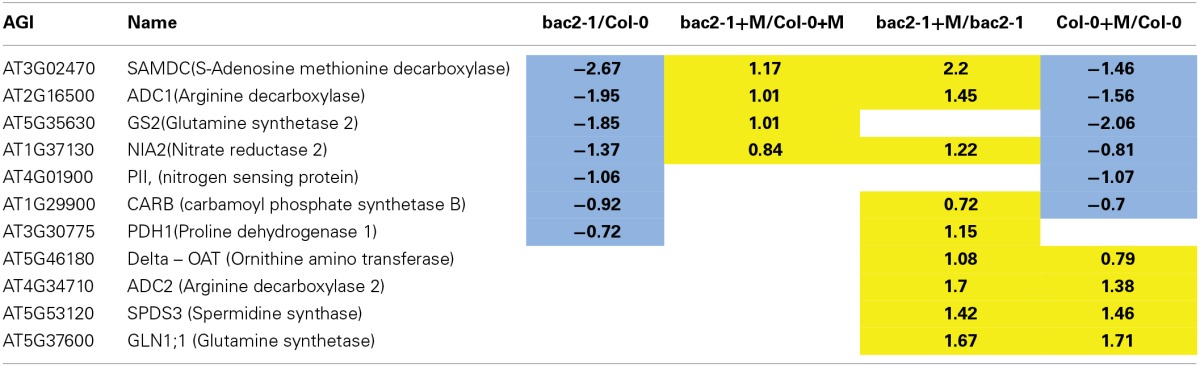
**Amino acids and arginine metabolism related genes differentially expressed in *bac2-1***.

*Detailed list of amino acids and arginine metabolism related genes which are differentially expressed in bac2-1. Negative log2 ratios are shown on a blue background, positive ones on a yellow background. Biological functions of genes were determined by amiGO Gene Ontology Software (Boyle et al., 2004)*.

By using a GO term finder (Boyle et al., 2004), we generated a list of amino acid and nitrogen transporters whose gene expression was modified in at least one of our experiments (Table 3). Amongst this list, *AAP1* is downregulated in *bac2-1* but its gene expression is highly induced during stress in *bac2-1*. AAP1 is a transporter of the neutral amino acids glutamate, alanine, and histidine, and is expressed in roots and developing embryos (Lee et al., 2007). The regulation of expression of other transporter genes known to be involved in water and salt stress responses is also different between WT and *bac2-1*. Such genes include *NRT1.5*, a nitrate transporter (Chen et al., 2012), *OCT5* an organic cation transporter (Küfner and Koch, 2008) and *PHT3;1*, an inorganic phosphate MCF (Zhu et al., 2012). Expression of these genes is always regulated differently in *bac2-1* compared to WT.

**Table 3:**
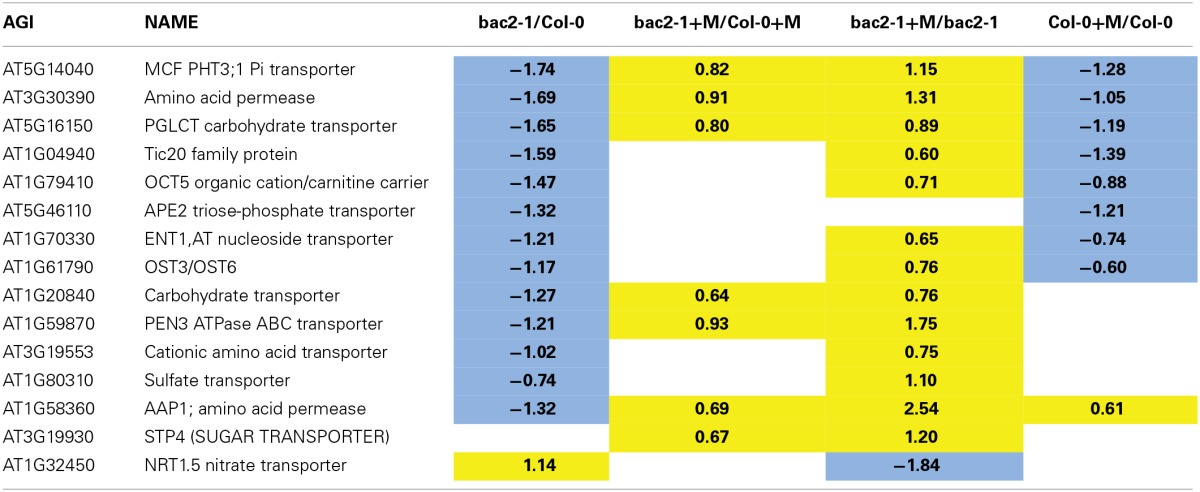
**Carrier genes differentially expressed in *bac2-1***.

*Detailed list of carrier genes that are differentially expressed in bac2-1. Negative log2 ratios are shown on a blue background, positive ones on a yellow background. Biological functions of genes were determined by amiGO Gene Ontology Software (Boyle et al., 2004)*.

## Discussion

### BAC2 expression modulates arginine degradation and urea production

Arginine is synthesized in plants from glutamate in the chloroplast. Arginine is a precursor of several molecules, such as amino acids, polyamines or urea (Slocum, 2005; Witte, 2011). When *BAC2* expression is high in *BAC2*-*OE* lines, arginine content is low. This lower arginine content could be caused by an increase of arginine transport to mitochondria where arginine catabolism takes place.

The pathway of arginine catabolism in mitochondria involves the arginase enzyme. In Arabidopsis two genes, *ARGAH1* and *ARGAH2*, encode mitochondrial arginases (Brownfield et al., 2008; Flores et al., 2008). Two-week-old *argah* mutants and overexpressing lines have less arginine than the wild type (Shi et al., 2013). Arginase activity leads to the production of ornithine and urea. Urea that accumulates strongly in *BAC2-OE* lines could be the product of arginine degradation by arginase activity after it has been transported into mitochondria by BAC2. Ornithine and citrulline, two molecules that are products of arginine catabolism, also accumulate in *BAC2-OE* plants. Urea accumulated mainly in aerial parts of *BAC2*-*OE* lines, which is consistent with the expression of *ARGAH2* in leaves from the seedling stage onwards (Brownfield et al., 2008; see also expression data from BAR eFP Browser shown on Supplementary figure 3).

Urea can be imported into the cell by plasma membrane located DUR3 and TIP type transporters (Liu et al., 2003; Kojima et al., 2007). Urea sensing leads to specific gene expression, leading to its degradation by urease into CO_2_ and ammonium. Ammonium in turn can be assimilated as a nitrogen source (Mérigout et al., 2008; Witte, 2011). Urease activity is dependent on a nickel (Ni) fixing activating subunit (Witte, 2011). The *in vitro* growth medium (MS medium) used in our experiments does not contain Ni and the agar used to solidify the growth medium is not known to be a source of Ni. When plants such as Arabidopsis are grown on Ni deficient soil supplemented with urea, urea accumulates because degradation is slow (Mérigout et al., 2008; Witte, 2011; Arkoun et al., 2012). As urea accumulates in leaves of *BAC2*-*OE* lines, the urease enzyme might not be active enough to eliminate the excess urea produced by an increased arginine mitochondrial transport and catabolism. Our data showed that a higher urease activity was detected in the presence of Ni in the medium and consequently, urea content decreased in *BAC2-OE* plants. Excess accumulation of urea in *BAC2*-*OE*, is thus due to both induced arginine catabolism and ineffective urea degradation in limiting Ni experimental conditions.

### BAC2 has a unique function in plants

BAC2 belongs to the BAC family of eukaryotic mitochondrial basic amino acid carriers which have similar biochemical functions that can complement the *arg11 S. cerevisiae* mutant, deficient in the ARG11 arginine and ornithine carrier (Palmieri et al., 1997; Catoni et al., 2003; Fiermonte et al., 2003; Hoyos et al., 2003; Palmieri et al., 2006a,b, 2011; Palmieri and Pierri, 2010). The biological role of BAC in plants is certain to be different because of specific differences in basic amino acid metabolism, especially the fact that arginine is synthesized another organelle, the chloroplast (Slocum, 2005).

Plants do not rely on a urea cycle as animals do although some photosynthetic organisms not from the plant lineage, such as diatoms, are thought to use a diverted urea cycle for nitrogen assimilation and recycling (Allen et al., 2011). Plants can metabolize arginine for nitrogen recycling during germination and during senescence but as seen in this study they rely on active urease enzymes. Arginine is also a precursor of molecules that have been found associated with stress responses, such as polyamine and NO (Witte, 2011). Although not as well documented as for mammals, plant mitochondria are known to produce the signaling molecule, nitric oxide (NO). This NO production is arginine-dependent (Guo and Crawford, 2005) and *Arabidopsis thaliana* arginase mutants that are unable to catabolize arginine produce more NO than wild type (Flores et al., 2008). Under the action of arginine decarboxylase, arginine can also be transformed into agmatine, a precursor in polyamine biosynthesis, in a pathway that is regulated by stress in plants (Perez-Amador et al., 2002).

As *BAC2* is expressed during stress and senescence (Toka et al., 2010) and increases arginine catabolism *in planta*, we can hypothesize that arginine catabolism is modulated in these conditions by the BAC2-dependent transport to the mitochondria.

We found that BAC2 is needed for full recovery of leaf growth after stress. Possibly, BAC2 arginine transport and degradation in the plant mitochondria could play a role in nitrogen metabolism in eliminating excess arginine, recycling nitrogen and urea allowing the production of primary molecules. Alternatively, arginine availability, modulated by BAC2, for NO synthesis in the mitochondria or polyamine synthesis in the cytoplasm might be important for the response to and recovery from stress.

### *BAC2* function is required for proper gene expression

Given the importance of arginine as a basic amino acid at the crossroads of several pathways leading to the synthesis of stress-related molecules, we assessed the effects of the *bac2* mutation on gene expression.

The *bac2-1* mutant transcriptome was different than the WT transcriptome. The *bac2-1* mutant showed a specific response in hyperosmotic conditions and also a deregulation of gene expression in control conditions.

We identified TFs whose expression behave differently as they are upregulated in *bac2-1* in the absence of stress but are regulated similarly in both *bac2-1* and WT upon stress (*SZF1*, *WRK33*, *CZF1*, and *ERF5*). WKR33 and ERF5 are of particular interest since they are induced in Arabidopsis leaves during mild osmotic stress (Skirycz et al., 2010). They are also involved in plant responses to pathogens. ERF5 regulates chitin-response genes (Son et al., 2012) and ectopic overexpression of WRKY33 increases plant resistance to the plant pathogen fungus *Botrytis cinerea* (Zheng et al., 2006). ERF5 belongs to a TF family that activates expression of stress-related TF such as WRKY33 (Dubois et al., 2013).

Genes involved in arginine (*ADC1*, *CARB*) and polyamine (*SAMDC*) metabolisms are deregulated in *bac2*. Also, a few transporters of amino acids, nitrogen and phosphate, known to be regulated by stress behave differently in *bac2*.

Mutation of other mitochondrial carriers is known to have consequences on gene expression leading to specific metabolic pathway impairment in Arabidopsis. Mutation of the mitochondrial carrier *BOU* gene, for example, was shown to down-regulate photosynthetic genes and up-regulate abiotic stress response genes, leading to photorespiration inhibition (Eisenhut et al., 2013). Arabidopsis overexpressing *PHT3;1*, a mitochondrial phosphate carrier, showed changes in expression of genes involved in gibberellin metabolism during salt stress leading to a higher sensitivity to salt stress (Zhu et al., 2012).

Altogether our results indicate that both in control conditions, when *BAC2* expression is low, and in stress conditions, when *BAC2* expression is high, BAC2 plays an indirect role in the expression of a specific set of genes. Control of gene expression in Arabidopsis, optimized growth after stress, and arginine and urea homeostasis are all dependent upon *BAC2* arginine carrier expression and function.

## Author contributions

Séverine Planchais, Cécile Cabassa, Anne-Marie Justin, Iman Toka, and Pierre Carol were involved in designing and performing mutant analysis, molecular genetics and gene expression experiments. They interpreted and co-wrote the corresponding sections. Séverine Planchais, Jean-Pierre Renou, and Pierre Carol conceived the experiments, interpreted the results and wrote the sections regarding transcriptome analysis. Cécile Cabassa, Séverine Planchais, Arnould Savouré, and Pierre Carol conceived and interpreted the amino acid and urea analysis experiments. Arnould Savouré and Pierre Carol conceived and interpreted the stress experiments. Séverine Planchais, Pierre Carol, Cécile Cabassa, and Arnould Savouré co-wrote the final versions of the paper.

### Conflict of interest statement

The authors declare that the research was conducted in the absence of any commercial or financial relationships that could be construed as a potential conflict of interest.
