# Supplementary material for: BASIC AMINO ACID CARRIER 2 gene expression modulates arginine and urea content and stress recovery in Arabidopsis leaves
Source: Front Plant Sci. 2014 Jul 16;5:330. doi: 10.3389/fpls.2014.00330 (PMC4099941; doi:10.3389/fpls.2014.00330)
Supplement: Supplementary file 1 [file DataSheet1.PDF]

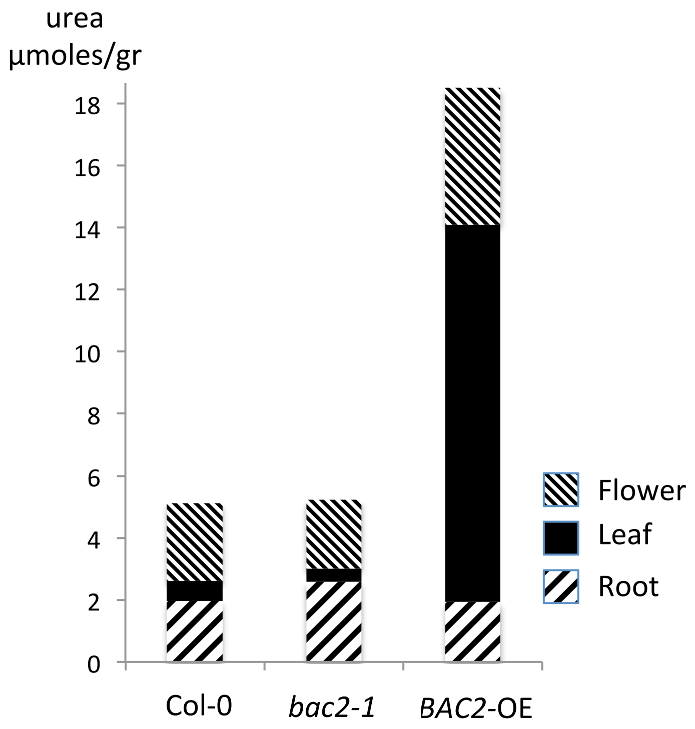

Supplementary figure 1: urea accumulation in three-week-old plants.

Total and organ-specific urea accumulation (in micrograms per gram of fresh weight) in three-weeks-old plants grown in soil, n=3.

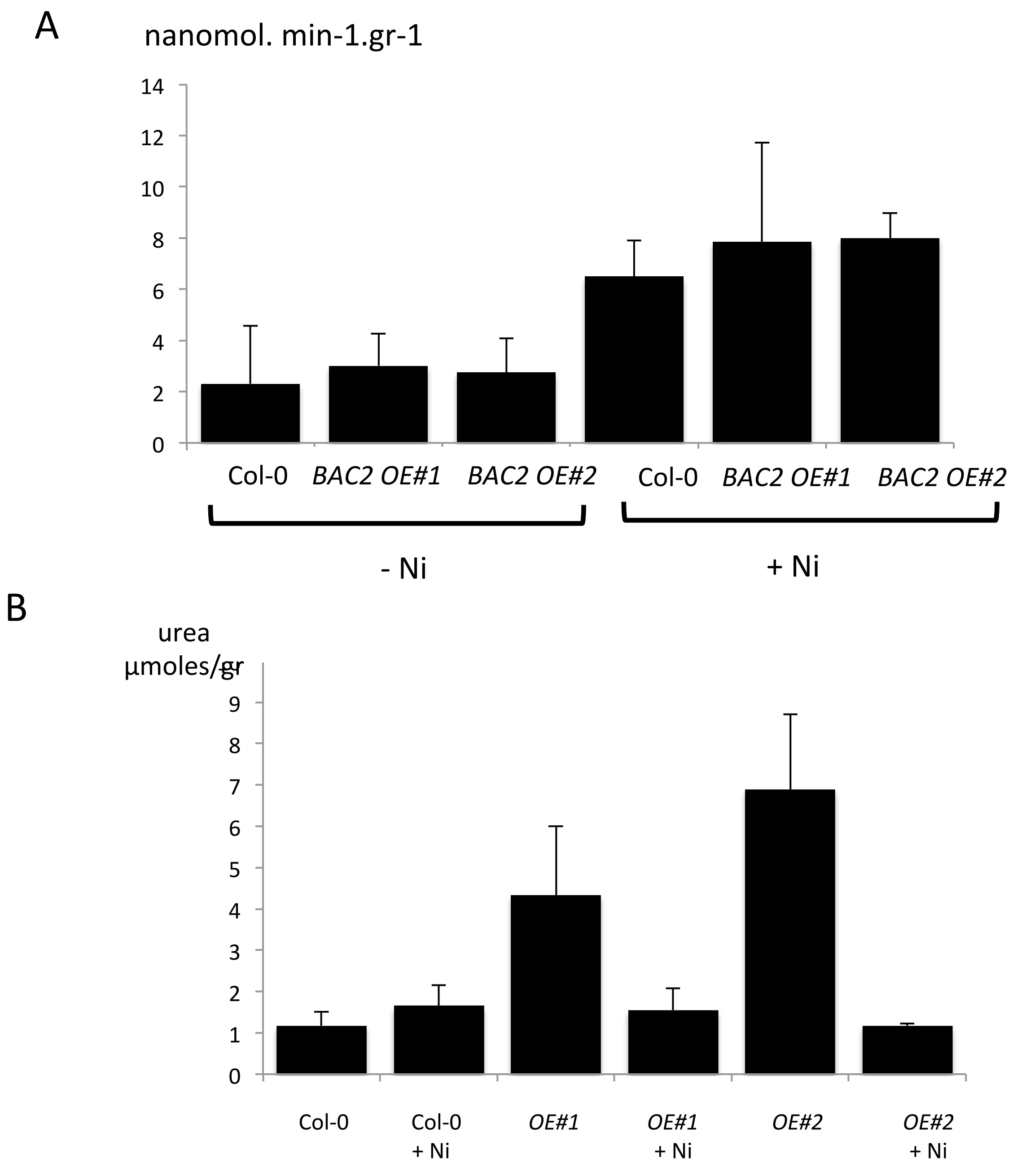

Supplementary figure 2 . Effect of Nickel on urea accumulation and urease activity

(A) Urease activity (measured as urea catabolised per minute and gram of fresh weight) increases when plants are grown in the presence of nickel in the growth medium (5 micromolar NiCl).

(B) Urea accumulation in 12-day-old seedlings

Urea accumulation in seedlings grown in MS medium devoid or supplemented with 5 micomolar nickel (NiCl). *BAC2 OE* plants show a urea accumulation that similar to that of wild-type control.

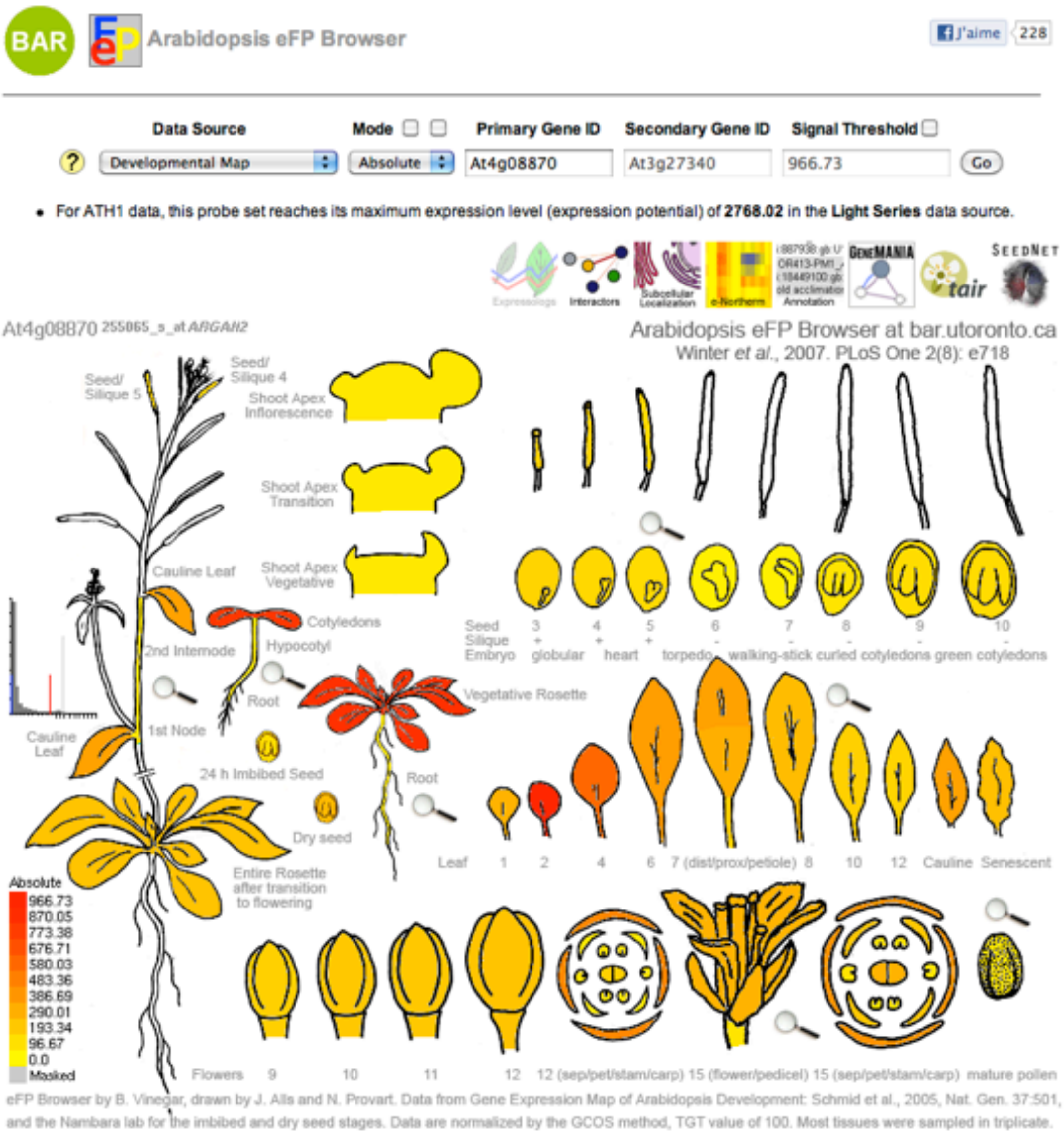

Supplementary figure 3: Relative *ARGAH2* (At4g08870) gene expression. Aerial (leaves) organs: 846 +/- 99; roots: 44 +/- 6. Taken from BAR website http://bar.utoronto.ca/efp/cgi-bin/efpWeb.cgi.
